# Intermuscular two-incision technique for implantation of the subcutaneous implantable cardioverter defibrillator: a 3-year follow-up

**DOI:** 10.1007/s10840-023-01478-z

**Published:** 2023-01-20

**Authors:** Federico Migliore, Raimondo Pittorru, Enrico Giacomin, Pietro Bernardo Dall’Aglio, Pasquale Valerio Falzone, Emanuele Bertaglia, Sabino Iliceto, Dario Gregori, Manuel De Lazzari, Domenico Corrado

**Affiliations:** 1 Cardiology, Department of Cardiac, Thoracic, Vascular Sciences and Public Health, University of Padua, Via Giustiniani 2, 35121 Padova, Italy; 2 Statistics, Department of Cardiac, Thoracic, Vascular Sciences and Public Health, University of Padua, Padova, Italy

**Keywords:** Complications, Intermuscular two-incision technique, Subcutaneous implantable cardioverter defibrillator

## Abstract

**Purpose:**

The aim of the present study was to evaluate the outcome of patients underwent subcutaneous implantable cardioverter defibrillator (S-ICD) implantation with the intermuscular (IM) two-incision technique during 3-year follow-up.

**Methods:**

the study population consisted of 105 consecutive patients (79 male; median 50 [13–77] years) underwent S-ICD implantation with the IM two-incision technique. The composite primary end point of the study consisted of device-related complications and inappropriate shocks (IAS). Secondary end points included the individual components of the primary end point, death from any cause, appropriate therapy, major adverse cardiac events, hospitalization for heart failure, and heart transplantation.

**Results:**

According to the PRAETORIAN score, the risk of conversion failure was classified as low in 99 patients (94.3%), intermediate in 6 (5.7%).Ventricular fibrillation was successfully converted at ≤65 J in 97.4% of patients. During a median follow-up of 39 (16–53) months, 10 patients (9.5%) experienced device-related complications, and 9 (8.5%) patients reported IAS. Lead-associated complications were the most common (5 patients, 4.7%), including 2 cases of lead failure (1.9%). Pocket complications were reported in 2 patients (1.9%). Extra-cardiac oversensing (3.8%) represented the leading cause of IAS. No T-wave oversensing episodes were recorded. Twelve patients (11.4%) experienced appropriate shocks. Eight patients (7.6%) died during follow-up. IAS or device-related complications did not impact on mortality.

**Conclusions:**

The overall device-related complications and IAS rates over 3 years of follow-up were 9.5% and 8.5%, respectively. According to our findings, the IM two-incision technique allows for optimal positioning of the device achieving a low PRAETORIAN score with a high conversion rate. IM two-incision technique allows low incidence of pocket complications, shifting the type of complications towards lead-related complications, which represent the most common complications. The IM two-incision technique would not seem to impact the occurrence of IAS. Management of complications are safe without impact on the outcome.

**Supplementary Information:**

The online version contains supplementary material available at 10.1007/s10840-023-01478-z.

## Introduction

The subcutaneous implantable cardioverter defibrillator (S-ICD) has become a recognized effective alternative to the transvenous ICD (TV-ICD) [1, 2]. The S-ICD allows to reduce the risk of systemic infection and lead failure, which are the most common complications of TV-ICD often requiring surgical revision. The intracardiac leadless configuration makes the S-ICD a preferrable choice mostly in young with inherited arrhythmogenic diseases patients [3] with a long life expectancy, in patients at high risk for infection, in subjects with inadequate vascular access or with previous infection/failure of TV-ICD in whom pacing is not needed [1, 2]. The traditional S-ICD implantation technique, which involves three incisions and insertion of the pulse generator (PG) under the subcutaneous tissue, has significantly changed over time. A new technique that uses two incisions and an intermuscular (IM) pocket for the PG between the serratus anterior and the latissimus dorsi muscles has been introduced and is currently widely adopted [4, 5]. Optimal implantation of the S-ICD requires minimizing the amount of adipose tissue between the coil and the sternum and between the PG and the thorax [5–8]. This results in less fat interposition between the PG and the chest and a low PRAETORIAN score (<90), reducing shock impedance and a high probability of effective defibrillation [8]. However, data on long-term outcome of the IM two-incision implantation technique are lacking. The aim of the present study was to evaluate the long-term outcome of patients underwent S-ICD implantation with the IM two-incision technique.

## Material and methods

### Study population

The study population of this retrospective single-center study included 105 consecutive patients (79 male; median, 50 [13–77] years) who received *de novo* S-ICD implantation with the IM two-incision technique for the prevention of sudden cardiac death, between November 2014 and November 2021.

Baseline clinical characteristics, electrocardiographic data, indication for implantation, electrocardiogram (ECG) screening, and technical device characteristics were collected. All S-ICD implantations were performed by experienced operators. The local ethics committee approved the study protocol and all patients provided written consent to be enrolled in the registry.

### S-ICD implantation technique

Before implantation, all patients underwent screening for S-ICD eligibility using the Boston Scientific manual ECG screening tool or the automated screening tool based on the surface ECG limb lead recording over the left and/or right parasternal regions to simulate the three S-ICD sensing vectors. To be eligible for S-ICD implantation, at least one ECG lead (I, II, or III) must satisfy the template (at any gain) in both erect and supine postures. All ECGs screening were reviewed by two experienced electrophysiologists blinded to patients, clinical presentation, and outcome. When there was disagreement, the ECG for that patient

was adjudicated by a third independent observer. The implantation procedure was performed in an electrophysiology laboratory under standard sterile conditions and general, local anesthesia with conscious sedation or ultrasound-guided serratus anterior plane block. Antibiotic prophylaxis was administered 1 h before the procedure. The IM two-incision technique was used for implantation, as previously reported in detail [4], Briefly, the IM two-incision technique abandons the superior parasternal incision and consists of creating an IM pocket (between the anterior surface of the serratus anterior muscle and the posterior surface of the latissimus dorsi muscle) for the PG rather than a subcutaneous pocket using anatomical landmarks. The position of the lead and PG relative to the heart silhouette is checked by fluoroscopy. An incision is made along the inframammary crease at the anterior edge of the latissimus dorsi. When the latissimus dorsi anterior edge is exposed, the pocket is created by blunt dissection between the superior surface of the serratus anterior muscle and the posterior surface of the latissimus dorsi muscle such that the PG can be placed into the virtual anatomical space between the two muscles (Supplementary Fig. 1). When the serratus anterior is reached, it is important to recognize the change in the fiber pathway (horizontal vs vertical) to preserve the muscular fascia may and thereby minimize bleeding. A 2-cm horizontal incision at the level of the xiphoid process (xiphoid incision) is made in the direction of the pocket incision. The distal tip of the electrode insertion tool which is used to create subcutaneous tunnels in which the electrode is placed, is inserted at the xiphoid incision and tunnelled laterally until the distal tip emerges at the device pocket. Correct placement of the tip of the lead at the required sternomanubrial location is confirmed digitally. The suture sleeve is secured to the fascia. The proximal end of the lead is inserted into the connector port in the device header of the S-ICD and the screw set is tightened. Thus, the device is located in the IM pocket and anchored to the fascia to prevent possible migration by using conventional nonabsorbable suture material. Particular attention is paid to ensure that the PG is placed posterior and inferior to the incision. Finally, the two muscles (serratus anterior and latissimus dorsi) are sutured using a conventional absorbable suture. Then, after device setup, the two incisions (xiphoid and pocket incisions) are closed using an intradermal suture.

### Defibrillation testing

After the procedure, defibrillation testing (DT) was performed after induction of ventricular fibrillation (VF) by 50-Hz stimulation. The DT was considered successful if the device detected and terminated VF using ≤65 J shock. In case the first shock failed with standard polarity and was effective at the same energy with reverse polarity without the need for implant revision, the test was considered successful. On the contrary, in the case of a 65 J shock failure and further successful test either after implant revision or at >65 J, the test was considered failed. The decision to perform post-implant DT was at the discretion of the implanting physician considering also the clinical condition of the patient. In patients who did not undergo DT a synchronized 10 J shock in sinus rhythm was considered.

### Chest radiograph analysis

A chest X-ray (both anterior–posterior and lateral view) was obtained the day after the procedure to confirm stable lead and PG position. Quality was judged adequate if the complete coil and PG were visualizable. The PRAETORIAN score was calculated according to a three-step approach as reported previously in detail [7]. Based on the final score, three risk categories were defined: (1) low risk of conversion failure: PRAETORIAN score of <90 points; (2) intermediate risk of conversion failure: PRAETORIAN score between ≥ 90 and <150 points; (3) high risk of conversion failure: PRAETORIAN score of ≥ 150.

### Device programming

In all patients, the device programming features included two tachyarrhythmia detection zones: (1) the shock-only zone, in which detection and therapy were based on rate only and (2) an additional “conditional zone,” in which a morphology analysis algorithm was applied in addition to rate. Rate cutoffs were individualized for each patient based on clinical indications. The sensing vector (primary, secondary, or alternate) was automatically selected by the device at the time of implantation and optimized during supine and upright positions.

### Follow-up and endpoints

All patients were followed up at 1 month and every 3 to 6 months thereafter. At these visits, patients’ clinical conditions, S-ICD interrogations, and complications were assessed. Remote device monitoring was also used. The composite primary end point of the study consisted of device-related complications and inappropriate shocks (IAS). Complications included pocket infection, lead infection requiring system extraction; pocket hematoma that led to drainage, incomplete wound healing, skin erosion of PG or electrode, blood transfusion, or prolongation of hospitalization; device-related thrombotic events; pneumothorax or hemothorax that led to intervention or prolongation of hospitalization; cardiac perforation or tamponade; lead repositioning or replacement; and other complications related to the lead or generator that required medical or surgical intervention. A lead failure was considered if it did not meet its performance specifications or otherwise perform as intended and required removal or abandonment because judged nonfunctional [9]. An ICD shock was classified as inappropriate when triggered by anything other than ventricular tachycardia or VF above the programmed rate zone, including supraventricular arrhythmias (SVT), cardiac/noncardiac oversensing, or device or lead malfunction. Cardiac oversensing was defined as T-wave oversensing (TWOS), QRS oversensing, P-wave oversensing or oversensing due to a low amplitude signal, and other/combined types of cardiac oversensing. Noncardiac oversensing was defined as any kind of oversensing due to noncardiac causes (e.g., electromagnetic interference and myopotentials). Secondary end points included the individual components of the primary end point, death from any cause, appropriate ICD therapy, major adverse cardiac events, and hospitalization for heart failure, heart transplantation. An appropriate shock was defined as a therapy delivered because of correctly diagnosed shockable rhythm. Captured S-ECG tracings from all shock episodes stored in the S-ICD were obtained and reviewed for details by two electrophysiologists. Episodes of inappropriate therapy were reviewed and verified with the Boston Scientific Technical support team.

### Statistical analysis

Categorical differences between groups were evaluated by using the chi-square test (*X*^2^) or the Fisher exact test as appropriate. Continuous variables were expressed as mean ± standard deviation (SD) or median with 25–75% for normally distributed and skewed variables, respectively, and compared with the Student’s *t*-test or the Wilcoxon rank sum test, as appropriate. Cumulative incidence functions were estimated and plotted to account for competing risks between IAS, device-related complications and death. Univariable effects of covariates for risk of IAS and device-related complications were estimated using the Fine & Gray approach. Univariable and multivariable analysis of predictors of mortality was based on a proportional hazard model. Non-linearity of effects was considered by introducing a restricted cubic-spline transform, with its significance assessed by Wald test. The proportionality of hazard assumption was assessed via a visual inspection of Schoenfeld residuals. The effect of IAS and device-related complications on mortality were included in the model as time-dependent covariates. Multivariable models were selected using the Bayesian Information Criterion evaluated in a forward fashion among predictors resulted significant at univariable analysis. A two-tailed p value <0.05 was considered statistically significant. Multivariable model performance was assessed using the Somer’s D_xy_ estimated via 1000 Bootstrap replicates. All statistical analysis were performed with SPSS (IBM SPSS Statistics Version 24.0.0, Armonk, NY) and the R System.

## Results

### Baseline variables

Baseline clinical characteristics are reported in Table 1. Thirty-three (31.4%) patients were implanted for secondary prevention. One patient was less than 18 years of age. At the time of ICD implantation, 66 (62.8%) patients were being treated with β-blocker and 12 (11.4%) were receiving an antiarrhythmic agent. Left ventricular (LV) dysfunction (ejection fraction ≤ 50%) was present in 46 (43.8%) patients. The reason for S-ICD placement was the presence of previous TV-ICD (patients underwent lead extraction for infection or lead failure) in 32 (30.4%) patients. In the remaining patients, the choice of implanting an S-ICD rather than a TV-ICD was at the discretion of the physician, which was based on clinical indications.

**Table 1:** Baseline clinical characteristics of the study population

Characteristics	*N*=105
Male	79 (75)
Age, years	50 (13–77)
BMI (kg/m^2^)	24 (22–26)
Secondary prevention	33 (31.4)
History of AF	9 (8.5)
Hypertension	29 (27.6)
Kidney disease (GFR < 60ml/min/1.73m^2^)	8 (7.6)
Dyslipidemia	26 (24.7)
Diabetes mellitus	14 (13.3)
Previous transvenous ICD	32 (30.4)
ECG characteristics	
Sinus rhythm	96 (91.4)
QRS duration, ms	110 (70–120)
PQ interval, ms	160 (114–235)
First AVB (PQ interval > 200 ms)	12 (11.4)
LV ejection fraction	53 (17–76)
Underlying disease	
Dilated cardiomyopathy	14 (13.3)
Ischemic heart disease	25 (23.8)
Hypertrophic cardiomyopathy	18 (17.1)
Arrhythmogenic cardiomyopathy	17 (16.1)
Brugada Syndrome	15 (14.3)
Long QT syndrome	3 (2.8)
Myocarditis	7 (6.6)
Idiopathic VF	4 (3.8)
Others	2 (1.9)
Medication at implant	
Beta-blockers	66 (62.8)
Antiarrhythmic agents	12 (11.4)
Diuretics	30 (28.5)
ACE-inhibitors or ARBs	44 (41.9)
Statin	33 (31.4)
Antiplatelets	23 (21.9)
Anticoagulants	16 (15.2)

*AF* atrial fibrillation, *ARBs* angiotensin receptor blockers, *AVB* atrioventricular block, *BMI* body mass index, *ECG* electrocardiogram, *GFR* glomerular filtration rate, *ICD* implantable cardioverter defibrillator, *LV* left ventricular, *VF*, ventricular fibrillation. Values are expressed as number/total (%) of patients or median (25th–75th percentile)

### ECG screening

The primary sensing vector was the most compatible (*n*=55, 52.4%), followed by the secondary vector (*n*=42, 40%) and the alternate vector (*n*=8, 7.6%). There were no cases with adjudication disagreement.

### S-ICD implant characteristics

**Fig. 1:**
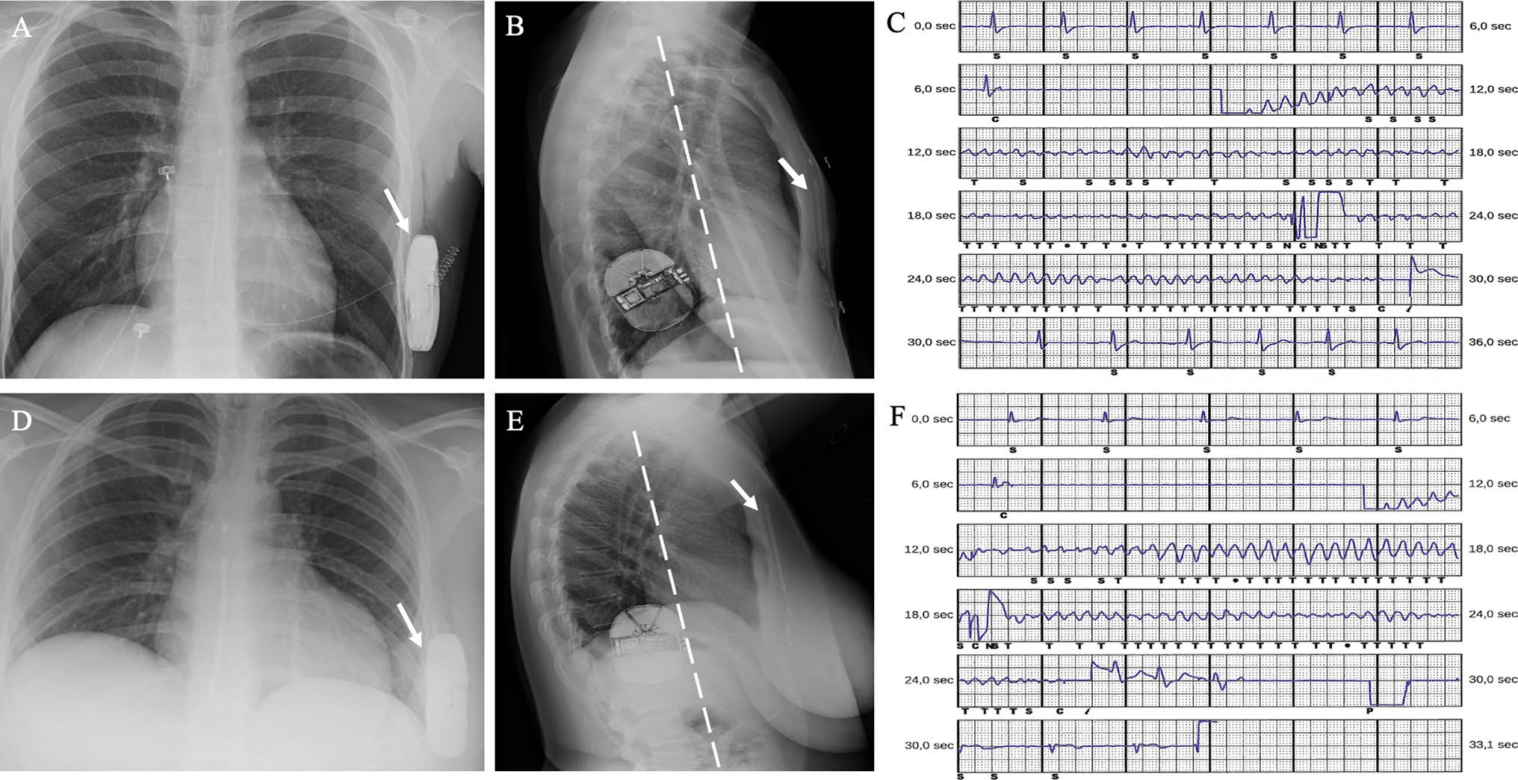
An example of optimal intermuscular pocket implantation in a patient with a BMI<25 kg/m^2^ (**A**, **B**) and in patient with a BMI >25 kg/m^2^ (**D**, **E**). There is a paucity of adipose tissue in the subcoil and sub-generator space (white arrows), and the generator is posterior to the midline (dashed white line). The PRAETORIAN score is <90 in both cases. Ventricular fibrillation successfully converted at ≤65J with standard polarity in both cases (**C**, **F**)

Baseline technical device characteristics are reported in Table 2. The procedure was performed under general anesthesia in 38 (36.2%) patients, local anesthesia with sedation in 29 (27.6%), and with ultrasound-guided serratus anterior plane block in 38 (36.2%) patients. The average procedure time (“skin to skin”) was 65 ± 18min. A postoperative chest radiography confirmed stable device and lead location in all patients. The S-ICD generator was on or posterior to the mid-axillary line in all patients. The distance between the generator and the thoracic wall was less than 1 generator width in all patients. According to the PRAETORIAN score, the risk of conversion failure was classified as low in 99 patients (94.3%), intermediate in 6 (5.7%). DT was performed in 77 (73.3%) patients. Twenty-eight patients did not undergo DT because of the presence of intracardiac thrombi in the left atrial appendage (*n* = 2) or in the LV apex due to prior myocardial infarction (*n* = 2), persistent atrial fibrillation with interruption of anticoagulation (*n* = 2), the presence of advanced cardiomyopathy with severe LV systolic dysfunction, and hemodynamic instability (*n* = 10), patient’s rejection (*n* = 1), and physician’s choice (*n* = 11). Ventricular fibrillation was successfully converted at ≤65 J in 97.4% (75/77) patients with standard polarity (Fig. 1). In the two patients in whom the ≤65 J shock failed, a second shock at 70 J was effective. These two patients had a PRAETORIAN score <90, a body mass index (BMI) <25 kg/m^2^, and the implant was performed under general anesthesia. Overall, median shock impedance was 68 Ohm (56–79). There was no difference in ≤65J shock impedance in patients with and without BMI ≥ 25 kg/m^2^). The mean time from VF induction to effective shock delivery was 16 ± 3 seconds. Of the 28 patients who did not undergo DT, 22 patients undergo synchronized 10 J shock in sinus rhythm with a median impedance of 64 Ω (55–74). No intra-procedural complications occurred. Dual-zone programming for tachyarrhythmia detection was selected in all patients and the SMART Pass® filter was activated in 91 patients (86.6%) after implantation.

**Table 2:** S-ICD implant characteristics

Implant characteristics	*N*=105
S-ICD model	
A209	13 (12.4)
A219	88 (83.8)
Cameron	4 (3.8)
Lead model	
3501	71 (67.7)
3401	28 (26.6)
3010	6 (5.7)
Lead position	
Left parasternal	96 (91.4)
Right parasternal	9 (8.6)
Programmed sensing vector	
Primary	55 (52.4)
Secondary	42 (40)
Alternate	8 (7.6)
Defibrillator testing attempted	77 (73.3)
VF conversion at ≤65J	75/77 (97.4)
Shock impedance, ohm	68 (56–79)
S-ICD programming	
Conditional shock zone (beats/min)	210 (200–230)
Shock zone (beats/min)	250 (210–250)

*S-ICD* subcutaneous implantable cardioverter defibrillator, *VF* ventricular fibrillation. Values are expressed as number/total (%) of patients or median (25th–75th percentile)

### Follow-up and primary end point

**Fig. 2:**
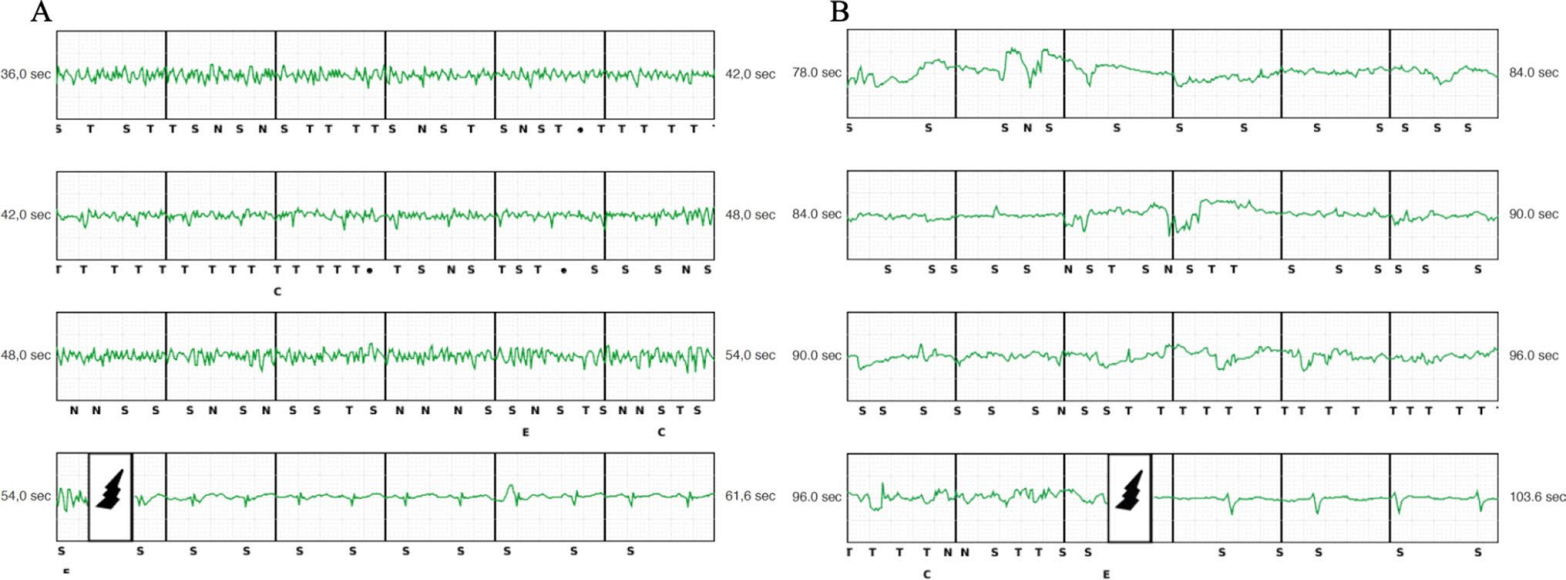
Extra-cardiac oversensing leading to inappropriate shock due to myopotential (primary vector) during effort in a patient with arrhythmogenic cardiomyopathy (**A**) and due to artifacts (primary vector) (**B**). In the latter case, the lead (3501) was removed for lead failure. No evidence of led fracture. Note the high-amplitude artifacts in combination with low signal amplitude

The median duration of follow-up was 39 (16-53) months. Eighteen patients (17%) experienced at least one complication. Nine patients (8.5%) received a total of 11 IAS (Fig. 2A and Supplementary Fig. 2). Ten patients (9.5%) experienced at least one device-related complication (Supplementary Fig. 3). One patient experienced both device-related complication (lead failure) and inappropriate shocks (noise due to lead failure). Device-related complications and reasons for IAS are reported in detail in Table 3. Among device-related complications, lead-associated complications were the most common (5 patients, 4.7%) including lead dislodgment (2 patients, 1.9%), lead failure (2 patients, 1.9%), and lead infection (1 patient, 0.9%). Extra-cardiac oversensing (4 patients, 3.8%) represented the leading cause of IAS (Fig. 2) following SVT (2.8%), especially atrial fibrillation (n=2). No IAS due to TWOS were recorded. Patients experienced IAS presented the following underlying cardiac disease: arrhythmogenic cardiomyopathy (*n*=2; reason of IAS: myopotentials), hypertrophic cardiomyopathy (n=2; reason of IAS: SVT, low-amplitude signal oversensing). Brugada syndrome (*n*=1; reason of IAS: repetitive premature ventricular beats); ischemic cardiac disease (*n*=1; reason of IAS: noise due to lead failure, Fig. 2B); dilated cardiomyopathy (*n*=1; reason of IAS: left ventricular assist device interference); myocarditis (*n*=1; reason of IAS: SVT); long QT syndrome (*n*=1; reason of IAS: SVT). We did not observe lead dislodgment in patients with IAS. Patients who experienced IAS due to SVT, cardiac oversensing or extra-cardiac oversensing underwent successful device reprogramming, including changing the sensing vector (*n*=4), catheter ablation (*n*=1), optimization of medical treatment (*n*=3), and device replacement for lead failure (*n*=1). At the time of the IAS episode, the SMART Pass® filter was found activated in 8 patients out of 9. A total of 8 device including both generator and lead were extracted with simple manual traction in 7 patients and using non-powered mechanical sheath in 1 patient. The reasons of device removal were pocket infection (*n*=2), lead failure (*n*=2), pocket erosion (*n*=1), ineffective therapy (*n*=1), and heart transplantation (*n*=2). Overall, S-ICD removal and TV-ICD re-implantation was required 4 patients (3.8%). The reasons were: infection (*n*=2), pocket erosion (*n*=1), and ineffective therapy (*n*=1). No patient had the device removed because of a perceived need for antitachycardia pacing or the necessity of pacing or cardiac resynchronization therapy. Premature cell battery depletion requiring device replacement was recorded in 2 patients (1.9%). No PG dislodgement, no late (>24 h) lead dislodgment, and no discomfort or systemic infection were observed during follow-up.

**Table 3:** Type of complications during follow-up

	*n* = 105
Patients experienced at least 1complication	18 (17)
Device-related complications requiring reintervention	10 (9.5)
Pocket-associated complications	2 (1.9)
Erosion	1 (0.9)
Infection	1 (0.9)
Lead-associated complications	5 (4.7)
Lead dislodgment (within 24 h)	2 (1.9)
Lead failure †	2 (1.9)
Lead infection	1 (0.9)
Others complications	
Premature cell battery depletion requiring device replacement	2 (1.9)
Ineffective therapy	1 (0.9)
S-ICD removal for complications and TV-ICD re-implantation*	4 (3.8)
Patients experienced inappropriate shocks	9 (8.5)
Reason for inappropriate shock	
Atrial fibrillation/supraventricular tachycardia	3 (2.8)
Cardiac oversensing	2 (1.9)
Repetitive premature ventricular beats	1 (0.9)
Low-amplitude signal oversensing	1 (0.9)
Extra-cardiac oversensing	4 (3.8)
Myopotentials	2 (1.9)
LVAD interference	1 (0.9)
Noise due to lead failure	1 (0.9)

*S-ICD* subcutaneous implantable cardioverter defibrillator, *TV-ICD* transvenous ICD, *LVAD* left ventricular assist device

^†^Model 3501 in both cases

^*^Pocket erosion (*n*=1), infection (*n*=2), ineffective therapy (*n*=1). Values are expressed as number/total (%) of patients

There was no significant difference between patients who did and did not have complications during follow-up with regard to clinical and device characteristics (Supplementary Table 1).

### Lead failure

**Fig. 3:**
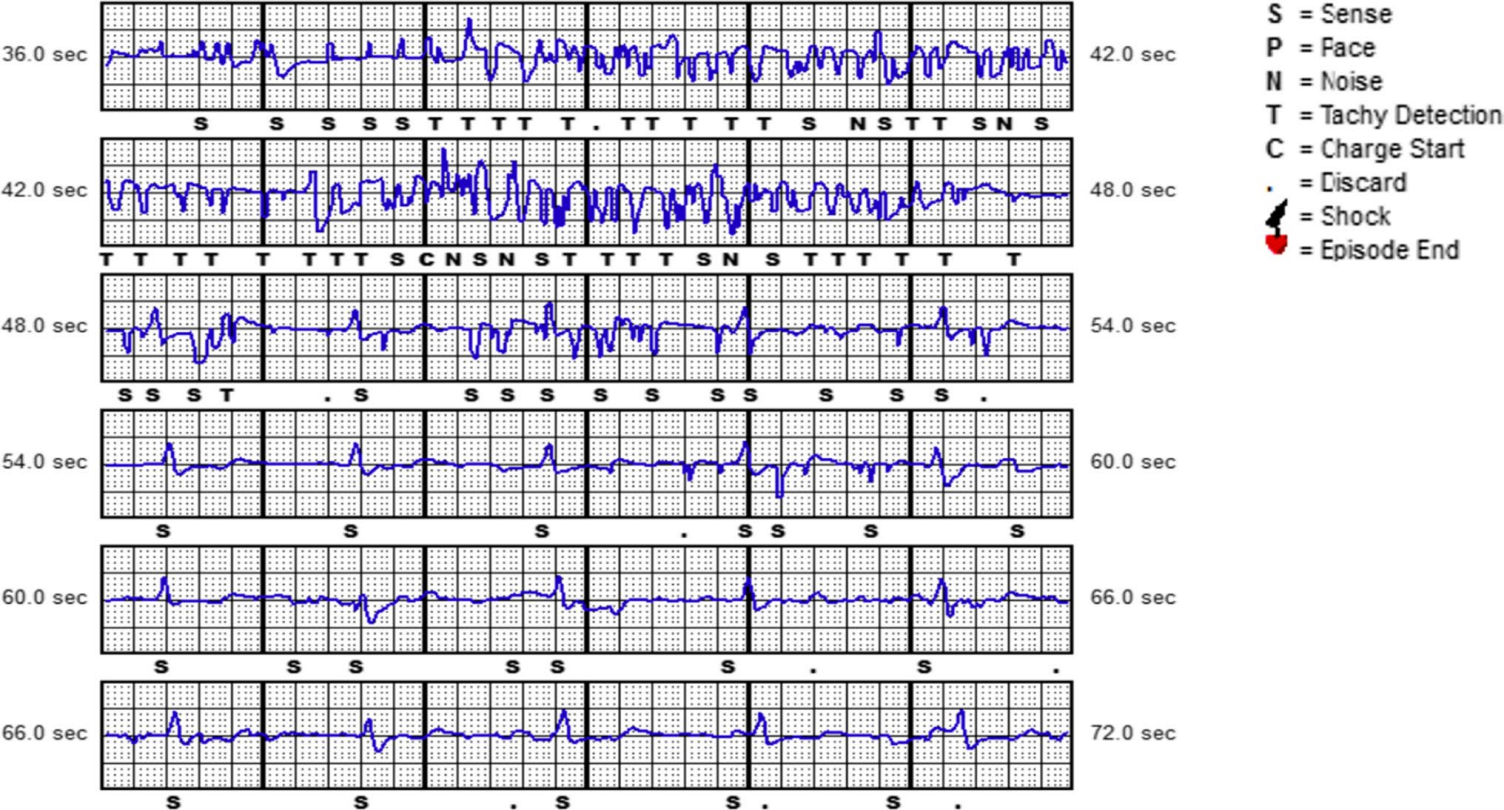
The presence of artifacts in a patient with 3501 lead (primary vector) extracted for lead failure. No evidence of led fracture

We reported two cases of lead failure consisting of sense B node issue (proximal sensing electrode), requiring device replacement. Both patients had a 3501 model lead. The first patient experienced IAS at rest 2 years after the implant, due to high-amplitude artifacts in combination with baseline shifts and sudden drops in signal amplitude in primary sensing vector (Fig. 2B). The artifacts were not reproducible. Secondary vector was not a valid sensing vector. Thus the system was extracted and reimplanted with a new S-ICD. The follow-up was uneventful. In the second patients we recorded an untreated episode at rest, due to high deflection noise on primary vector, 2 years after the implant (Fig. 3). The artifacts were reproducible manipulating the pocket. The system was extracted and reimplanted with a new S-ICD. The follow-up was uneventful. No lead fractures also due to recent Boston Scientific Recalls EMBLEM S-ICD Subcutaneous Electrode (Model 3501) were observed [10].

### Secondary end points

Twelve (11.4%) patients received a total of 30 appropriate shocks during follow-up. Eight patients (7.6%) died because of cardiac death due to refractory heart failure (*n*=6) or non-cardiac death (*n*=2) and 6 patients (6.6%) underwent rehospitalization for heart failure. Five patients (9%) underwent left ventricular assist device (LVAD) implantation due refractory heart failure, 5 (9%) patients underwent heart transplantation. There were no documented deaths associated with the S-ICD complications.

### Predictors of complications

There was no significant difference between patients who did and did not have S-ICD complications both device-related requiring surgical revision and IAS with regard to baseline clinical characteristics, S-ICD indication (Table 4).

**Table 4:** Predictors of inappropriate shocks, device-related complication, and mortality

Variable	Mortality				DRC			IAS	
	HR	(95% CI)	*P* value	HR	(95% CI)	*P* value	HR	(95% CI)	*P* value
Univariable analysis									
Ischemic heart disease	8.34	1.91–36.41	0.005	1.71	0.32–9.17	0.53	0.38	0.05–2.85	0.35
Kidney disease	5.28	1.06–26.35	0.043	1.82	0.18–18.9	0.62	1.44	0.19–10.8	0.72
Diabetes mellitus	15.32	3.54 – 66.21	0.001	1.19	0.13 – 11.0	0.88	–	–	–
Hypertension	5.23	1.24–22.01	0.024	2.71	0.56–13.0	0.21	0.71	0.15–3.33	0.67
LVEF*	0.22	0.06–0.84	0.026	0.99	0.93–1.05	0.65	1.02	0.98–1.06	0.41
Inappropriate shocks	0.007	–	0.844						
Device-related complications	0.002	–	0.864						
Multivariable model									
Diabetes mellitus	15.32	3.54–66.21	0.001						

*DRC* device-related complications, *IAS* inappropriate shocks, *LVEF* left ventricular ejection fraction

^*^The effect for LVEF is based on a non-linear term and presented in the range < 50% where effect is linear.

### Predictors of mortality

Univariable and multivariable analyses for predictors of mortality during follow-up are shown in Table 4. Univariable predictors of mortality included the presence of underlying ischemic heart disease (HR 8.34; 95% CI 1.91–36.41; *P* = 0.005), kidney disease (HR 5.28; 95% CI 1.06–26.35; *P* = 0.043), diabetes mellitus (HR 15.32; 95% CI 3.54–66.21; *P*= 0.001), hypertension (HR 5.23; 95% CI 1.24–22.01; *P*= 0.024), and left ventricular ejection fraction (HR 0.22; 95% CI 0.006–0.84; *P* = 0.026). In the multivariable model, only diabetes mellitus remained significant independent predictor of mortality (HR 15.32; 95% CI 3.54-66.21; P= 0.001). The presence of previous IAS or device-related complication was not predictors of mortality.

## Discussion

In this study, we evaluated the “real-word” long-term outcome of patients underwent S-ICD implantation with the IM two-incision technique. The composite primary end point of the study consisted of device-related complications and IAS. We focused on the type of complications, their management, possible predictors and the impact of complications on mortality during the follow-up. The main findings are:

I. the IM two-incision technique allows for optimal positioning of the device achieving a low PRAETORIAN score (<90) in most cases (94.3%) with a high conversion success rate at 65 J (97.4%) without pulse generator adjustments and with a low median impedance value independent to body mass index;
II. 9.5% of patients experienced device-related complications requiring reintervention. Pocket complications were relatively low suggesting the potential role of the IM two-incision technique. Instead, lead related complications were the most common (4.7%);
III. Management of all device-related complications was safe and effective, requiring mostly device removal. Lead removal required simply traction in the most of cases. No device-related deaths were observed;
IV. 8.5% of patients received at least one IAS despite the IM technique, new generation device with SMART Pass® filter in most cases and device programing with high rate cutoff. This because the extra-cardiac oversensing (3.8%) represented the leading cause of IAS. No IAS due to TWOS were recorded;
V. Although the relatively high incidence of complications, they did not impact on survival during follow-up.

### PRAETORIAN score, DT in S-ICD using intermuscular two-incision technique: which features are potential advantages ?

Clinical experience and computer modelling showed the highest detrimental effects on defibrillation thresholds by incremental fat under either the S-ICD lead coil or between the S-ICD can and the chest, and anterior positioning of the can [6, 7, 11, 12]. These findings prompted to develop the PRAETORIAN score, a noninvasive tool based on chest X-ray images (posterior–anterior and lateral) post-implantation to assess the optimal S-ICD implant position. According to this score, patients with a low PRAETORIAN score (<90) are associated with a low risk of conversion failure [7]. The IM pocket is created between the latissimus dorsi muscle and the anterior serratus muscle, which invariably positions the S-ICD can posteriorly, deep and close to the chest, leading to fat reduction under the S-ICD generator acting positively on the second and third components of the PRAETORIAN score [7, 8, 12]. Furthermore, anatomical landmarks with IM technique ensure the right position of the PG in obese patients in whom a suboptimal position is more common [4, 6–8, 11, 12]. In the present study using IM combined with the two-incision technique a low PRAETORIAN score (<90) was achieved in most cases. In all patients the IM technique allows for posterior positioning of the device and a low distance between the can and the chest highlighting that the only variable component of consequence is the subcoil proximity to the sternum/rib. For these reasons, the IM technique is the technique commonly adopted since it was proposed by our study group in 2017 [4]. Predictors of DT failure are higher BMI, suboptimal device position and increased impedance [6–8, 11]. In our study population using IM two-incision technique, the successful defibrillation rate at ≤65 J was high (97.4%) without PG adjustments with a low median impedance value independent to BMI. This is in line with previous evidence demonstrating that combining IM two-incision technique yielded the lowest PRAETORIAN scores and shock impedance values, indicating optimal defibrillation system position and a high probability of effective defibrillation [8]. Despite the current guidelines recommend routine DT for S-ICD implantation, many implanting physicians defer DT for S-ICD. Recent studies reported a declining trend of DT during S-ICD implantation without impact on clinical outcome [13]. Defibrillation testing requires more resource utilization such as anesthesia support and is not without risk, albeit rare, in patients with hemodynamic instability. A correlation between 10 J shock impedance and 65 J defibrillation impedance during IM S-ICD implantation have been demonstrated [13]. Thus, the IM pocket technique combined with 10 J shock in sinus rhythm may be sufficient to predict and ensure the defibrillation efficacy of the S-ICD. In our study, of the 28 patients who did not undergo DT, 22 patients undergo synchronized 10 J shock in sinus rhythm with a median impedance of 64 Ω (IQR, 55–74). All these concepts mentioned above are hypothesis generating that IM two-incision technique may eliminate routine PRAETORIAN score and DT during S-ICD implantation in most cases in the future. Since moving forward the knowledge leveraged from the PRAETORAN score should be transformed into better implant techniques, a prospective trial is needed to evaluate its predictive power [14]. This trial will tell us more about the ability to perform an optimal implant and how this translates into successful DT, aiming to avoid DT.

### Intermuscular two-incision technique and complications: which potential advantages?

Early trials of S-ICDs demonstrated relatively high device-related complications rates, partly attributable to the learning curve of implantation [15, 16]. The most common complications are surgical complications and pocket-associated including infection, erosion, and bleeding [16–18]. The EFFORTLESS Registry study reported complication rate of 8.9% and 15.2% at 1 year and 5 years, respectively [16]. The most common complications were infection and erosion requiring system removal [16]. The PRAETORIAN trial showed comparable complication rates between S-ICD and TV-ICD, with subcutaneous devices presenting more surgical complications (especially pocket hematoma) and transvenous devices presenting more lead related complications [1]. Although, noteworthy and numerically similar to TV-ICD, S-ICD complications are easier to manage and present a favorable outcome profile, with no device-related deaths, although hospitalization and reinterventions are required [1]. In a recent large independent multicentered study, ELISIR Registry, the “real-world” device-related complications rate was 9.3% over a median follow-up time of 23 months [17]. Pocket-related complications were the most common, with pocket hematoma and infections representing the leading causes [17]. In our study including the largest single-center study population of S-ICD implanted exclusively with the IM two-incision technique, the complications rate was 9.5% during a long-term follow-up, which is in line with that reported in the ELISIR Registry [17]. However, if we focused on the type of complications, in our study, lead-associated lead complications were the most common, and the pocket-associated complications were relatively low. This can be explained by the fact that the IM technique provides a larger pocket in a more posterior left axillary region, additional layering, a virtual space between the device and the chest, resulting in a potential reduction in pocket-related complications, particularly the skin erosion [4, 5, 12].

According to our results during the 3-year follow-up, the rate of IAS remains substantial, occurring in 8.5% patients despite new generation devices and systematic SMART Pass® availability in most cases, device programming, and optimal positioning of the device and lead. These results are comparable to recent large studies [17, 18] and to those of the first-generation S-ICDs studies [15]. Therefore, the IM two-incision technique would not seem to impact the occurrence of IAS given the substantial rate of IAS observed in our study. However, if we focused on the causes of IAS, extra-cardiac oversensing represented the leading cause. We did not obverse IAS due to TWOS. This can be explained certainly by the SMART Pass® detection filter which attenuates cardiac oversensing and especially TWOS [2], but it could be also be due to the IM technique which may improve cardiac sensing [4, 12] shifting the type of complications towards extra-cardiac sensing. The higher rate of IAS reported in our study compared in the UNTOUCHED study [2] might be related to the differences baseline clinical characteristics, such as young age, “real-life” population with different underlying cardiac disease, including inherited cardiomyopathies, patients with LVAD and the different median duration of follow-up. Moreover one patient experienced IAS secondary to lead failure. Young patient with inherited cardiomyopathies [3] and patients with LAVD are associated with an increased risk of IAS. Moreover, during a longer follow-up, the occurrence of any events/situations or comorbidity (i.e., cardiac disease progression, lead failure, physical activity) could affect the risk of IAS occurrence (3, 20).

### Potential strategy to avoid inappropriate shocks

Possible strategies that may reduce IAS are proper pre-implant ECG screening, device programming (single- vs dual-zone programing with high rate cutoff) and software upgrade including the “SMART Pass.” Targeting a surface ECG R-wave amplitude >1 mV at implant may allow for better discrimination. Furthermore, it will also be important to track the sensed R-wave amplitude in various vectors on follow-up. Moreover, specific cardiac diseases as the arrhythmogenic cardiomyopathy are progressive disease characterized by R-wave amplitude decline during follow-up predisposing this population to possible cardiac and/or non-cardiac oversensing and subsequent inappropriate therapy. Consequently, it may be desirable to have at least 2/3 vectors suitable in S-ICD [3].

### Study limitations

Although this study is the largest study with the longest follow-up period which assessed the outcome of patients underwent S-ICD implant with the IM two-incision technique, there are some limitations. This is a retrospective single-center study. No direct comparison was made between the traditional technique and IM two-incision technique, but this goes beyond the aim of the present study. Certainly, these studies should be performed in the future and international cooperation and merging of databases are essential to obtain more insight into this subject. Even with this long follow-up, there were a relatively small number of events and this might have affected the identification of predictors. All procedures were performed by experienced operators, and therefore, our results may not be widely applicable in less experienced centers. However, opting for optimal S-ICD implantation in low/medium-volume centers with evolving S-ICD implant program, the IM two-incision technique may provide the optimal technique to achieve better outcome.

## Conclusions

According to our study, the IM two-incision technique allows for optimal positioning of the device achieving a low PRAETORIAN score in most cases with a high conversion rate. Pocket complications are relatively low suggesting the potential role of the IM technique shifting the type of complications towards lead-related complications, which are the most common. The IM two-incision technique would not seem to impact the occurrence of IAS given the substantial rate of IAS, mostly due to extracardiac over sensing, observed in our study. Management of all complications is safe without impact on the outcome.

## Supplementary Information

Supplementary Figure 1. Anatomical landmarks for the intermuscular two-incision technique (**A**)**.** An example of optimal intermuscular pocket implantation. S‐ICD generator is placed in the plane between the serratus anterior and latissimus dorsi muscles (**B**). (PNG 992 kb)

High Resolution Image (TIFF 2980 kb)

Supplementary Figure 2. Cumulative Incidence curve for inappropriate shocks during follow-up. IAS: inappropriate shocks. (JPG 248 kb)

Supplementary Figure 3. Cumulative Incidence curve for device-related complications during follow-up. DRC: device-related complications. (JPG 242 kb)

Supplementary Table 1 (DOCX 38 kb)

## Data Availability

Data available on request from the authors.
