# Supplementary figures and images for: Intermuscular two-incision technique for implantation of the subcutaneous implantable cardioverter defibrillator: a 3-year follow-up
Source: J Interv Card Electrophysiol. 2023 Jan 20;68(5):1109–19. doi: 10.1007/s10840-023-01478-z (PMC12317887; doi:10.1007/s10840-023-01478-z)

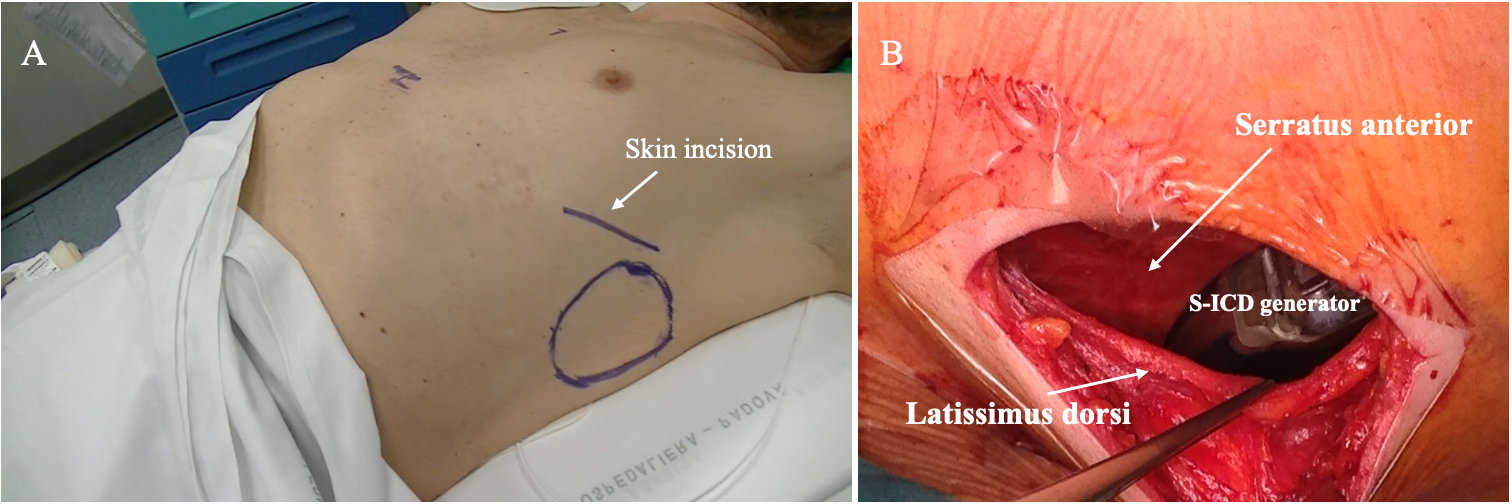

Supplement: Supplementary file 1 — Anatomical landmarks for the intermuscular two-incision technique (A). An example of optimal intermuscular pocket implantation. S‐ICD generator is placed in the plane between the serratus anterior and latissimus dorsi muscles (B). (PNG 992 kb) [file 10840_2023_1478_Fig4_ESM.png]

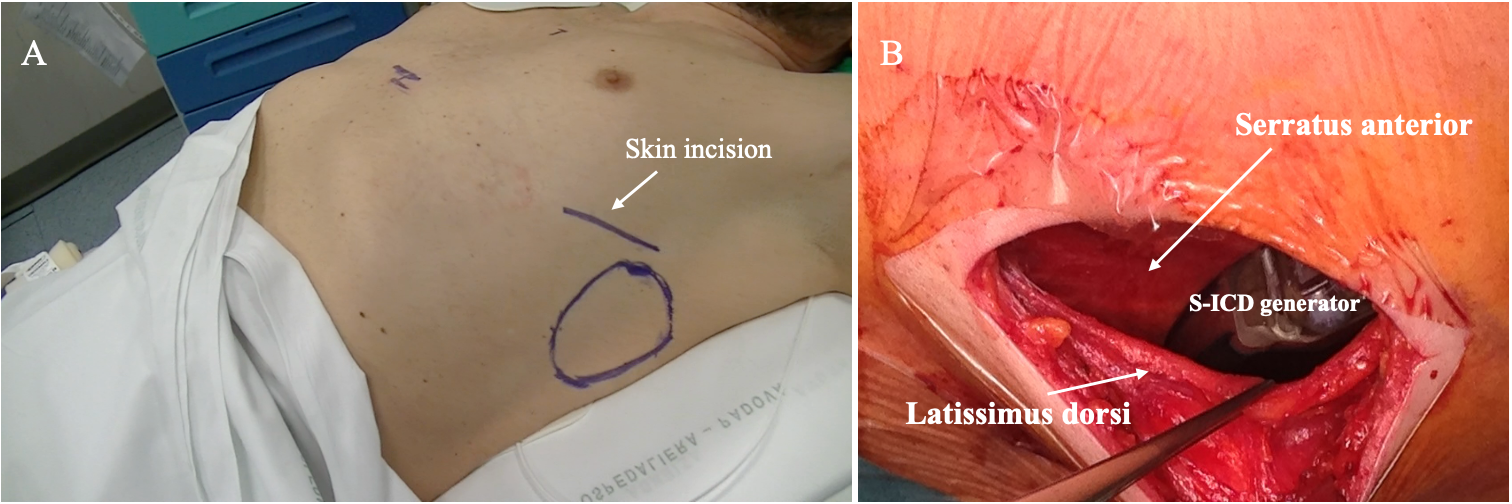

Supplement: Supplementary file 2 — High Resolution Image (TIFF 2980 kb) [file 10840_2023_1478_MOESM1_ESM.tiff]

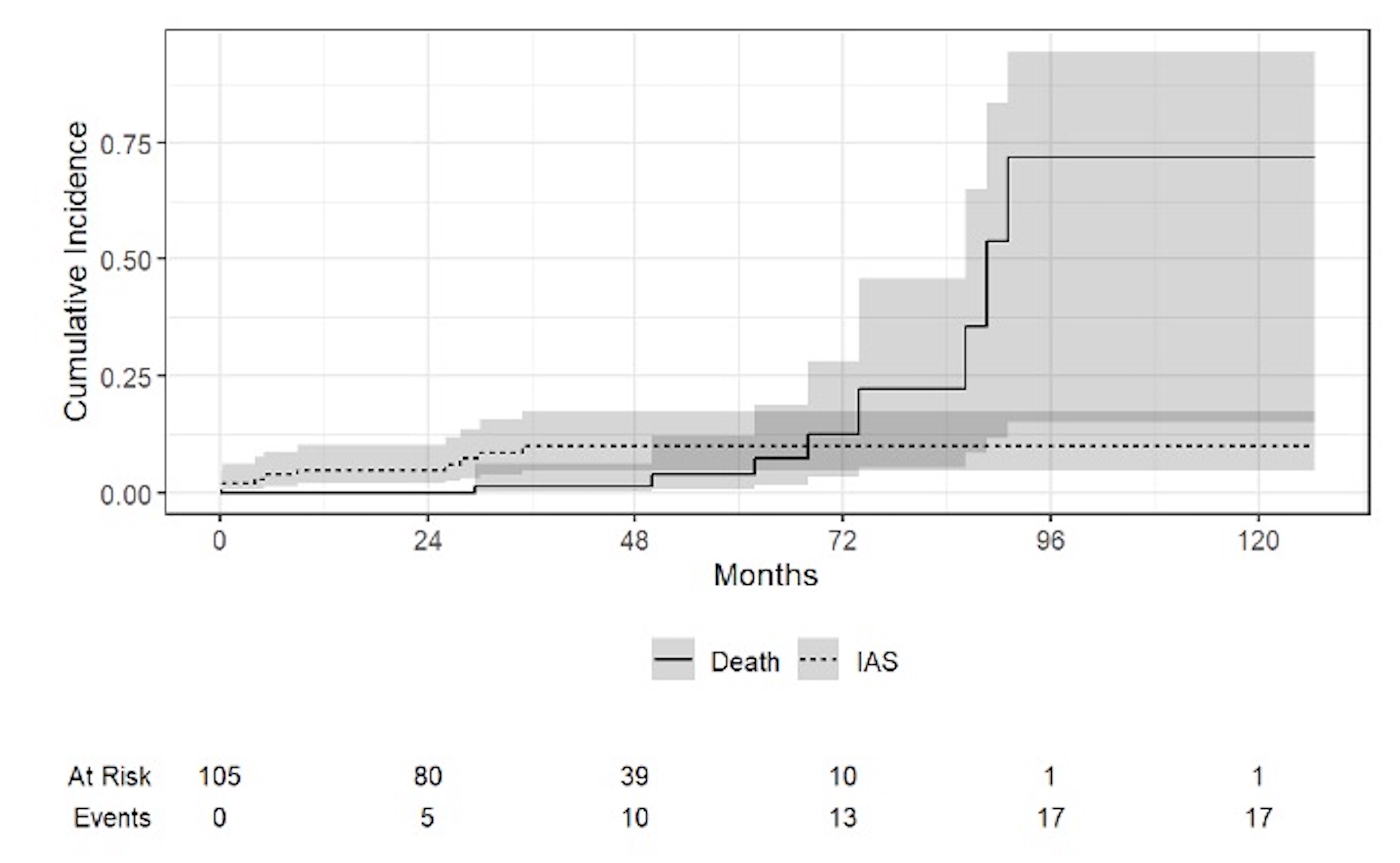

Supplement: Supplementary file 3 — Cumulative Incidence curve for inappropriate shocks during follow-up. IAS: inappropriate shocks. (JPG 248 kb) [file 10840_2023_1478_MOESM2_ESM.jpg]

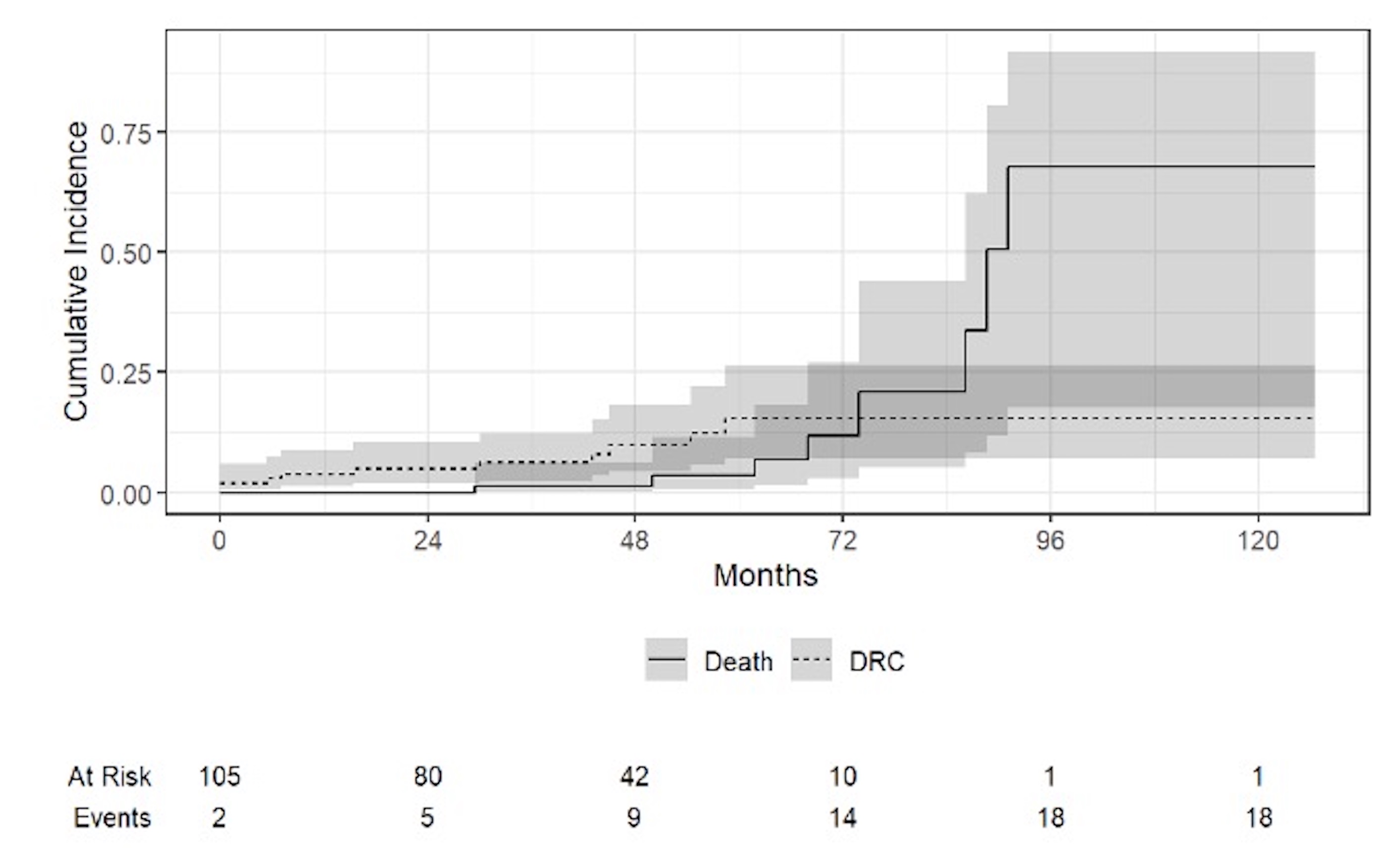

Supplement: Supplementary file 4 — Cumulative Incidence curve for device-related complications during follow-up. DRC: device-related complications. (JPG 242 kb) [file 10840_2023_1478_MOESM3_ESM.jpg]
