# Supplementary material for: Intermuscular two-incision technique for implantation of the subcutaneous implantable cardioverter defibrillator: a 3-year follow-up
Source: J Interv Card Electrophysiol. 2023 Jan 20;68(5):1109–19. doi: 10.1007/s10840-023-01478-z (PMC12317887; doi:10.1007/s10840-023-01478-z)
Supplement: Supplementary file 5 — (DOCX 38 kb) [file 10840_2023_1478_MOESM4_ESM.docx]

**Supplemental tables**

**Supplementary Table 1**. Clinical characteristics of patients with and without complications.

|  | No complications  (N= 87) | Complications  (N=18) | P value | |
| --- | --- | --- | --- | --- |
| Male | 64 (73) | 15 (83) | | 0.5 |
| Age (years) | 51 (39-57) | 46 (28-52) | | 0.3 |
| BMI (kg/m2) | 24 (22-26) | 23 (21-28) | | 0.7 |
| Secondary prevention | 28 (32) | 5 (27) | | 0.8 |
| History of AF | 7 (8) | 2 (11) | | 0.6 |
| Hypertension | 24 (27) | 5 (27) | | 0.8 |
| Kidney disease (GFR< 60 ml/min/1.73m2) | 6 (7) | 2 (11) | | 0.6 |
| Dyslipidemia | 21 (24) | 5 (27) | | 0.8 |
| Diabetes mellitus | 12 (13) | 2 (11) | | 0.8 |
| Previous transvenous ICD | 26 (29) | 6 (33) | | 0.8 |
| LV ejection fraction | 47 (15) | 51 (17) | | 0.3 |
| ECG characteristics |  |  | |  |
| Sinus Rhythm | 80 (92) | 16 (89) | | 0.3 |
| QRS duration (ms) | 100 (90-110) | 105 (96-121) | | 0.3 |
| PQ interval (ms) | 162 (150-180) | 170 (152-191) | | 0.2 |
| First grade AVB (PQ > 200ms) | 8 (9) | 4 (22) | | 0.3 |
| Implant characteristics |  |  | |  |
| S-ICD model A219 | 73 (83) | 15 (83) | | 0.4 |
| Lead model 3501 | 59 (67) | 12 (66) | | 0.8 |
| Left parasternal lead position | 79 (90) | 17 (94) | | 0.8 |
| S-ICD programming  Conditional shock zone (bpm) | 210 (200-220) | 210 (200-220) | | 0.5 |
| Shock zone (bpm) | 250 (250-250) | 250 (240-250) | | 0.6 |
| Death | 8 (9) | 0 | | 0.4 |

Abbreviations: AF, atrial fibrillation; AVB, atrioventricular block; BMI, body mass index; ECG, electrocardiogram; ICD, implantable cardioverter defibrillator; LV, left ventricular; S-ICD: subcutaneous implantable cardioverter defibrillator. Values are expressed as number/total (%) of patients or median (25th‐75th percentile).
